# Sludge reduction, nitrous oxide emissions, and phosphorus removal by oxic-settling-anaerobic (OSA) process: the effect of hydraulic retention time

**DOI:** 10.1007/s11356-024-34393-5

**Published:** 2024-07-20

**Authors:** Giorgio Mannina, Alida Cosenza, Daniele Di Trapani, Paulo Marcelo Bosco Mofatto

**Affiliations:** Engineering Department, Palermo University, Viale Delle Scienze, Bldg. 8, 90128 Palermo, Italy

**Keywords:** Wastewater treatment, Oxic-settling-anaerobic process, Greenhouse gas emission, Nutrient removal, Sludge reduction

## Abstract

This paper presents a study on reducing sewage sludge by an oxic-settling-anaerobic (OSA) pilot plant compared to the conventional activated sludge (CAS) process in view of resource recovery and moving towards plant carbon neutrality. The OSA plant was supplied with real wastewater and the anaerobic reactor was operated under two hydraulic retention times (HRT) (4 and 6 h). Greenhouse gas (GHG) emissions were monitored for the first time to determine the OSA process’s production mechanism. The results highlighted that under the lowest HRT (4 h), the removal efficiencies of COD and PO_4_^−^P, increased from 75 to 89% and from 39 to 50% for CAS and OSA configurations, respectively. The observed yield coefficient was reduced from 0.58 gTSS gCOD^−1^ (CAS period) to 0.31 gTSS gCOD^−1^ (OSA period). A remarkable deterioration of nitrification efficiency under OSA configuration was obtained from 79% (CAS) to 27% (OSA with HRT of 6 h). The huge deterioration of nitrification significantly affected the GHG emissions, with the N_2_O-N fraction increasing from 1% (CAS) to 1.55% (OSA 4 h HRT) and 3.54% (OSA 6 h HRT) of the overall effluent nitrogen, thus suggesting a relevant environmental implication due to the high global warming potential (GWP) of N_2_O.

**Graphical abstract:**

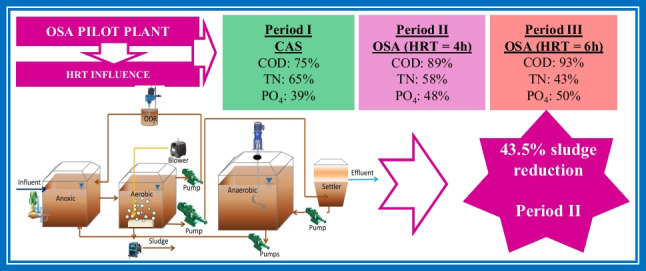

## Introduction

Nowadays, most wastewater treatment plants (WWTPs) are based on the well-known conventional activated sludge (CAS) layout. CAS process involves the biological conversion of biodegradable organic substrate, mediated by microorganisms, into energy and new cells, yielding an effluent compatible with the quality of the receiving water bodies. The produced sludge is rich in volatile nutrients and energetic potential, thus allowing its feasible usage for agricultural purposes and energy production (Martins et al. 2020). Nevertheless, the increasing production of excess sludge and the issues related to treatment and disposal operations represent one of the significant concerns regarding CAS systems (Mannina et al. 2023). Indeed, the costs for excess sludge treatment and disposal might account for up to 40–60% of the total operating costs in WWTPs based on the CAS process, thus generating a noticeable economic impact (Collivignarelli et al. 2019). Moreover, excess sludge disposal may cause secondary pollution (for the environment and human health) depending on the disposal method. In this light, reducing its production is a preferred option nowadays (Semblante et al. 2016a; Collivignarelli et al. 2021a). Moreover, the decrease of excess sludge production while using the residual sludge for agricultural purposes has become an imperative priority also in a circular economy perspective (Mannina et al. 2021a). Therefore, excess sludge minimization has two advantages: i. reduction of environmental pollution; ii. the decrease in WWTP’s operational costs is mainly related to sludge management (Mannina et al. 2022).

Several technologies have been suggested in the literature to reduce excess sludge production (chemical, physical, thermal, or biological) (Zhang et al. 2021). These technologies can be applied as post-treatment in the sludge line or as reduction sludge processes in the wastewater line (Coma et al. 2013; Morello et al. 2022). In addition, biological technologies have proven more sustainable than chemical processes (Collivignarelli et al. 2021b). Among the biological methods proposed in the literature, the oxic-settling-anaerobic (OSA) process is one of the most interesting approaches suggested (Morello et al. 2022). It offers several benefits, including efficiency, simplicity, and lower cost (Saby et al. 2003). Furthermore, the OSA process does not involve supplementary physical or chemical treatment with the final advantage of producing sludge that can be further valorised (Chen et al. 2003). The OSA process entails a change of a CAS system, by placing an anaerobic reactor in the return activated sludge (RAS) line (Chudoba et al. 1992). Excess sludge reduction in the OSA system takes place through the combination of several mechanisms (for example, uncoupled metabolism, biomass decay, destruction of extracellular polymeric substances (EPS)), which reduce the amount of produced sludge (Ferrentino et al. 2021). These mechanisms often overlap, and identifying the dominant one causing the reduction of sludge production is still challenging (Vitanza et al. 2019). Nevertheless, previous studies demonstrated that implementing the OSA system can promote a significant decrease in excess sludge production. Jiang et al. (2018) focused on the role of hydraulic retention time (HRT) in an OSA process, finding that the production of excess sludge can be reduced by 60% under HRT > 6 h. Nevertheless, some studies revealed that long HRTs in the anaerobic reactor might negatively affect the effluent quality (Semblante et al. 2016b; Jiang et al. 2018). Indeed, high HRT under low oxygen availability could affect nitrification, thus worsening the fundamental mechanisms of biological nitrogen removal (Cantekin et al. 2019). This aspect is prominent in plants operating biological nutrient removal, since the reduction of nitrification–denitrification efficiency may promote the production/emission of N_2_O, which is recognized as a crucial greenhouse gas (GHG) characterized by a more significant global warming potential (GWP) compared to CO_2_ (Mannina et al. 2018). On the other hand, previous studies emphasized that implementing the OSA process may promote phosphorus removal from the liquid phase also in systems not conceived for this purpose. Indeed, the alternation between aerobic and anaerobic conditions, which is typical of OSA systems, might favor the growth of phosphorus-accumulating organisms (PAOs), with the consequence of producing a phosphorus-rich sludge (Ye et al. 2008). This aspect might be of prominent interest since phosphorus recovery from the wastewater stream is becoming crucial in the circular economy and sustainability approach. In more detail: (i) P is crucial for fertilizer production; (ii) the significant abundance of P in wastewater may promote eutrophication if not removed; and (iii) P is primarily obtained from non-renewable sources such as phosphate rocks (Jupp et al. 2021; Zhang et al. 2022).

In view of these purposes, achieving a trade-off between sludge minimization and system performance in the OSA system, including nitrogen and P removal is assuming a pivotal role.

Moreover, to the best of the authors’ knowledge, applying the OSA process in literature studies mainly refers to pilot plants fed with synthetic wastewater. As the authors know, few studies have been conducted using real sewage and full-scale applications (Vitanza et al. 2019; Karlikanovaite-Balikci and Yagci 2019; Ferrentino et al. 2021). Indeed, how the variability of influent wastewater features may influence the OSA performance regarding sludge reduction is still an open issue in the literature (Karlikanovaite-Balikci and Yagci 2019). Further, the GHG emissions from the OSA process have not been monitored, and the potential contribution that such a system may provide compared to CAS systems must be assessed.

In this light, this study aims to gain insights into the performance of an OSA reactor by analyzing the sludge reduction efficiency, GHG emissions, and feasibility of P removal. In particular, an OSA pilot plant was monitored in the long term to assess the influence of HRT in the anaerobic reactor. The system was fed with real wastewater collected from the Palermo University (Italy) campus.

## Material and methods

### Description of plant layout

**Fig. 1:**
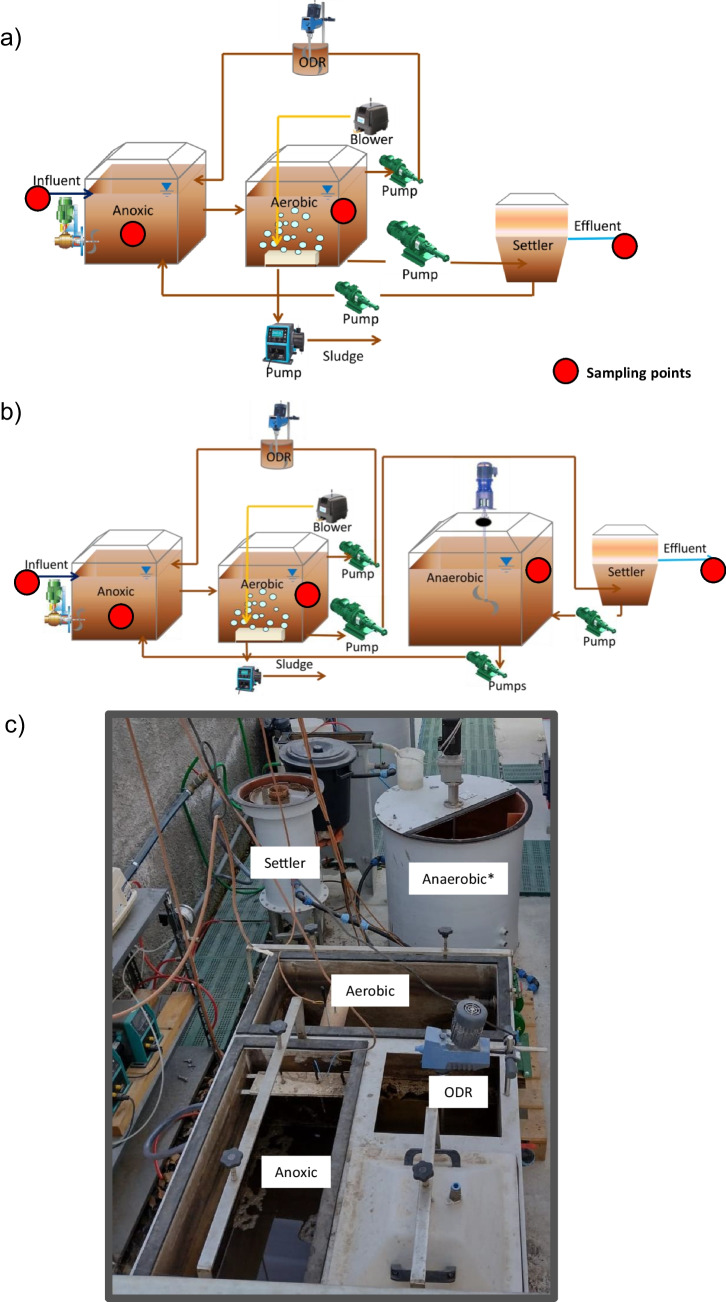
Representation of the pilot plant with the indication of sampling points: CAS system with pre-denitrification scheme (**a**) and CAS-OSA system with the anaerobic reactor in the sludge return line (**b**); pilot plant picture (**c**)

A pilot scale plant was realized at the Water Resource Recovery Facility of Palermo University (Mannina et al. 2021b). The system (Fig. 1) was realized as a CAS process in a pre-denitrification scheme, conceived for carbon and nitrogen removal. The units were one anoxic reactor (*V* = 110 L), followed by one aerobic reactor (*V* = 240 L), and a vertical settler (*V* = 46 L) for solids separation. An oxygen depletion reactor (ODR) (*V* = 53 L) was placed in the internal recycling line to minimize the oxygen load to the anoxic reactor. Moreover, one anaerobic side-stream reactor (ASSR) (*V* = 176 L for HRT = 4 h and *V* = 275 L for HRT = 6 h) was added to the RAS line to implement the OSA configuration, as showed in Fig. 1b. The influent wastewater was fed into the anoxic reactor per gravity using an electro-valve whose opening was controlled by water level sensors installed inside the anoxic reactor.

Real wastewater, collected from the Campus of Palermo University (Italy), was used for feeding the pilot plant, with an influent flow rate of 20 L h^−1^. Mannina et al. (2021b) described that the wastewater was collected by a pumping station and sent to the pilot plant via a pipeline. An 80 L h^−1^ (QR1) flow rate of mixed liquor was pumped from the aerobic to the anoxic reactor through the ODR (internal recycle) to enhance heterotrophic denitrification. A flow rate of 65 L h^−1^ was fed from the aerobic reactor to the final settler (*Q*_R2_), whilst a RAS flow rate equal to 45 L h^−1^ (*Q*_RAS_) was recycled from the bottom of the settler to the anoxic reactor. Since the features of the real wastewater were related to the activities of the campus canteen, authors expected slight variations; therefore, an equalization tank of 2 m^3^ was installed close to the campus canteen, while a 1-m^3^ tank was installed close to the pilot plant, thus providing an overall equalization volume of 3 m^3^. As shown in Fig. 1, the system was connected by sanitary progressive cavity pumps (Nova Rotors MN Series Progressive Cavity Pump). At the same time, the withdrawal was done by means of peristaltic pumps (Watson Marlow Qdos 30 Universal pump).

### Experimental campaign

The experimental campaign had a duration of 120 days and was split into three different periods, namely period I, period II, and period III. In period I, the pilot plant was managed as CAS configuration (53 days). In period II, a CAS-OSA layout was implemented by introducing an ASSR reactor in the RAS line characterized by an HRT of 4 h (duration: 38 days). Finally, in period III, the plant layout was the same as in period II, but the HRT of the anaerobic reactor was increased to 6 h (duration: 29 days). In view of maintaining a constant concentration of TSS in the aerobic reactor, the sludge was withdrawn and used for polyhydroxyalkanoates (PHA) production or used to produce compost, according to the project described by Mannina and Mineo (2023) and Mannina et al. (2021b), respectively. Table 1 summarizes the main features of the influent wastewater as well as the main operational parameters of the pilot plant throughout experiments (average values). On the other hand, the overall HRT of the system was assessed as the ratio between the overall volume (given by the sum of the anoxic, aerobic, and settling tanks) and the flow rate fed to the system.

**Table 1:** Influent wastewater characteristics and operational parameters of the systems

			Period I		Period II		Period III	
Parameter	Symbol	Units	CAS—control period		CAS-OSA (HRT = 4 h)		CAS-OSA (HRT = 6 h)	
			Average	SD	Average	SD	Average	SD
Total COD	TCOD	[mg L^−1^]	688	246	1463	387	1478	478
Soluble COD	sCOD	[mg L^−1^]	186	83	430	162	264	51
Total nitrogen	TN	[mg L^−1^]	35	12	36	5	38	10
Ammonium	NH_4_-N	[mg L^−1^]	28	8	28	9	26	9
Phosphate	PO_4_-P	[mg L^−1^]	4	2	8	2	13	10
Flow rate	Q_IN_	[L h^−1^]	20	0.4	18	2.5	18	0
Hydraulic retention time	HRT	[h]	22	0.4	25	3	24	0
Period duration		[d]	52	-	38	-	29	-
Average temperature	T	[°C]	11.6	1.7	13.8	2.3	18.6	1.9
Sludge retention time	SRT	[d]	27	11	41	14	23	9

*SD* standard deviation

### Analytical methods

The operational parameters, such as pH, oxidation–reduction potential (ORP), and DO, were acquired daily using dedicated probes coupled to a multimeter (WTW 3340).

Chemical oxygen demand (COD), ammonia nitrogen (NH_4_^+^-N), nitrate nitrogen (NO_3_^+^-N), nitrite nitrogen (NO_2_^+^-N), orthophosphate (PO_4_-P), total and volatile suspended solids (TSS and VSS, respectively) concentrations, biochemical oxygen demand (BOD), and total nitrogen (TN) were measured twice a week according to literature (APHA 2012). The sludge volume index (SVI) assessed the sludge settling features. Extracellular polymeric substances (EPS) and soluble microbial products (SMP) were extracted and measured according to literature (Le-Clech et al. 2006); proteins and carbohydrates were assessed according to Lowry et al. (1951) and DuBois et al. (1956).

The excess sludge produced daily (ΔX) [kgSS d^−1^] was evaluated as the sum of TSS in the effluent, the TSS of the wasted sludge and the TSS in the collected samples. Δ*X* included both a “primary” sludge (associated with the inert settleable solids in the influent wastewater) and a “secondary” sludge (or biological, related to bacterial growth). Primary sludge was evaluated considering only the daily amount of influent settleable suspended solids. The secondary sludge was assessed as the difference between Δ*X* and the primary sludge. It is worth noting that in our study the term “primary” sludge was introduced only to discriminate the sludge produced by the settleable solids contained in the influent wastewater from the biological one, since no primary clarifier was present in the system.

The observed yield coefficient (*Y*_obs_) was evaluated by dividing the TSS produced by the COD removed, in terms of cumulated mass (Eq. 1) (Gardoni et al. 2011).

1 $${Y}_{\text{obs}}=\frac{\Delta X}{{Q}_{i}\cdot \left({\text{TCOD}}_{\text{in}}-{\text{TCOD}}_{\text{out}}\right)} (\text{gTSS} {\text{gCOD}}^{-1})$$

where TCOD_in_ and TCOD_out_ are the inlet and outlet total COD concentrations (gCOD L^−1^), *Q*_*i*_ is the daily influent flow rate (L d^−1^), and Δ*X* is the excess sludge produced daily (gTSS d^−1^).

The *Y*_obs_ values were corrected to the standard temperature of 20 °C (*Y*_obs_,_20_) according to Vitanza et al. (2019) (Eq. 2).

2 $${Y}_{\text{obs} T}= {Y}_{\text{obs}, 20}*{\theta }^{\left(20-T\right)} (\text{gTSS }{\text{gCOD}}^{-1})$$

where *T* = temperature and *θ* = 1.029.

Indeed, according to the literature, biomass yield is highly affected by temperature and sludge age (Tchobanoglous et al. 2003; Coma et al. 2013). Therefore, the seasonal variations of temperature during experiments must be considered for the final calculation of the observed yield.

The biomass stoichiometric and kinetic parameters were evaluated through respirometric batch tests carried out at 20 °C as reported by Mannina et al. (2016). Specifically, the maximum growth rate (*μ*_*H*_), the endogenous decay coefficient (*b*_*H*_), the maximum yield coefficient (*Y*_*H*_), and the active fraction of heterotrophic biomass (*f*_XH_), as well as the maximum yield coefficient (*Y*_*A*_) and the maximum growth rate (*μ*_*A*_) of autotrophic biomass were assessed in agreement to literature (Capodici et al. 2016). In addition, during the respirometric tests, the oxygen utilization rate (OUR) was measured from the biomass oxygen consumption after spiking a readily biodegradable substrate (e.g., acetate for heterotrophic and ammonium chloride for autotrophic bacteria).

Dissolved and gaseous N_2_O concentrations were evaluated according to the procedure reported by Mannina et al. (2018) by using a gas chromatograph (GC) (Agilent 8860) with an electron capture detector (ECD) device.

The N_2_O emission factor (EF_N2O_) was assessed according to Tsuneda et al. (2005) (Eq. 3).

3 $${\text{EF}}_{\text{N}2\text{O}}= \frac{{{\text{N}}_{2}\text{O}-\text{N}}_{\text{g}}/{\text{HRT}}_{\text{hs}}+{{\text{N}}_{2}\text{O}-\text{N}}_{\text{d}}/\text{HRT }}{\text{TN}}$$

where N_2_O-N_g_ and N_2_O-N_d_ are respectively the gaseous and dissolved nitrous oxide concentration, HRT is the pilot-plant hydraulic retention time, HRT_hs_ is the retention time in the tank headspace, and TN is the concentration of total nitrogen in the influent flow.

## Results and discussion

### Pilot plant removal performances

**Fig. 2:**
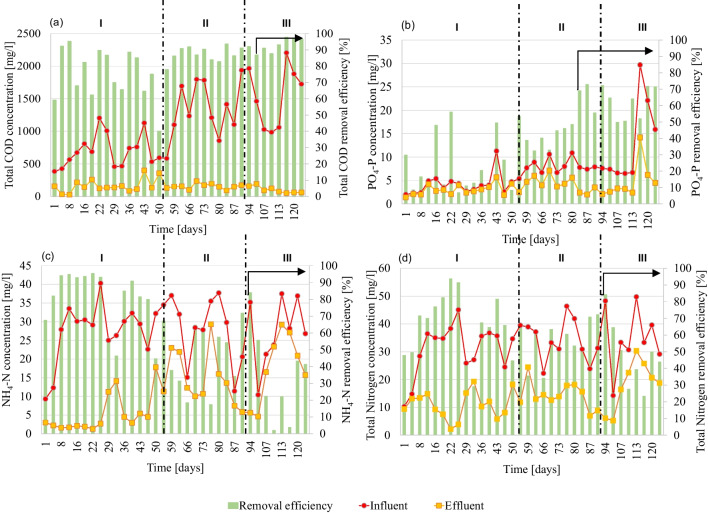
Pattern of influent, effluent and removal efficiency for total COD (**a**), PO_4_-P (**b**) NH_4_-N (**c**), and total nitrogen (**d**) for periods I (CAS), II (CAS-OSA, HRT = 4 h), and III (CAS-OSA, HRT = 6 h)

Figure 2 reports the trend profile of total COD, PO_4_-P, NH_4_-N, and total nitrogen concentrations throughout experiments, coupled with the associated removal efficiencies.

During the experimental campaign, the average pH in the reactors did not change significantly (7.8 ± 0.2, 7.8 ± 0.2, and 7.9 ± 0.3 in periods I, II, and III respectively). Dissolved oxygen (DO) in the anoxic, OSA and ODR reactors were equal to 0 mg L^−1^ in all the periods, while in the aerobic reactor was equal to 4.3 ± 1.9, 2.8 ± 1.2, and 2.7 ± 1.7 mg L^−1^ in periods I, II, and III, respectively. ORP of OSA reactor in periods II and III was equal to − 173.8 ± 68.8 and − 154.1 ± 80.6 mV.

As reported in Fig. 2, it is possible to notice a fluctuation in the influent wastewater quality, likely due to the different students’ habits during the experimental periods.

In period I, the average influent Total COD concentration was lower compared to the subsequent periods (688 mgCOD L^−1^ ± 246, 1463 mgCOD L^−1^ ± 386, and 1477 mgCOD L^−1^ ± 478 in periods I, II and III, respectively). This was likely connected to dilution because of rainy weather. In period I, the average COD removal efficiency was around 75% which was lower compared to period II (89% ± 4) and period III (93% ± 4). This result pointed out that COD removal was not influenced by the OSA configuration but mainly by the influent features. Moreover, the OSA configuration (periods II and III) also showed slightly higher removal efficiencies than previous studies. As an example, Martins et al. (2020) obtained 86% of COD removal using real wastewater with an OSA system characterized by an HRT of 12 h in the ASSR. The best result obtained in the present study compared to what achieved by Martins and co-workers could be related to the lower HRT in the ASSR, which likely prevented the biomass stress thus maintaining good efficiency in terms of COD removal.

Regarding PO_4_-P (Fig. 2b), a significant improvement in the removal efficiency occurred during OSA operation (for both periods II and III). Indeed, in period I, the average removal efficiency of PO_4_-P was 24%. In contrast, in periods II and III, the removal efficiency increased up to 50 and 61%, respectively. According to the literature, these results suggested that it might be possible the enhancement of phosphate accumulating organisms (PAOs) or denitrifying phosphate accumulating organisms (DPAOs) growth under OSA operation due to anaerobic conditions (Fazelipour et al. 2021). Indeed, the EPS destructuration, coupled with SMP release in the bulk liquid, could have provided the carbon source for PAO or DPAO organisms. Furthermore, these results have important implications in terms of the circular economy since the sludge produced in periods II and III is rich in phosphorus and could be further valorised as fertilizer.

**Fig. 3:**
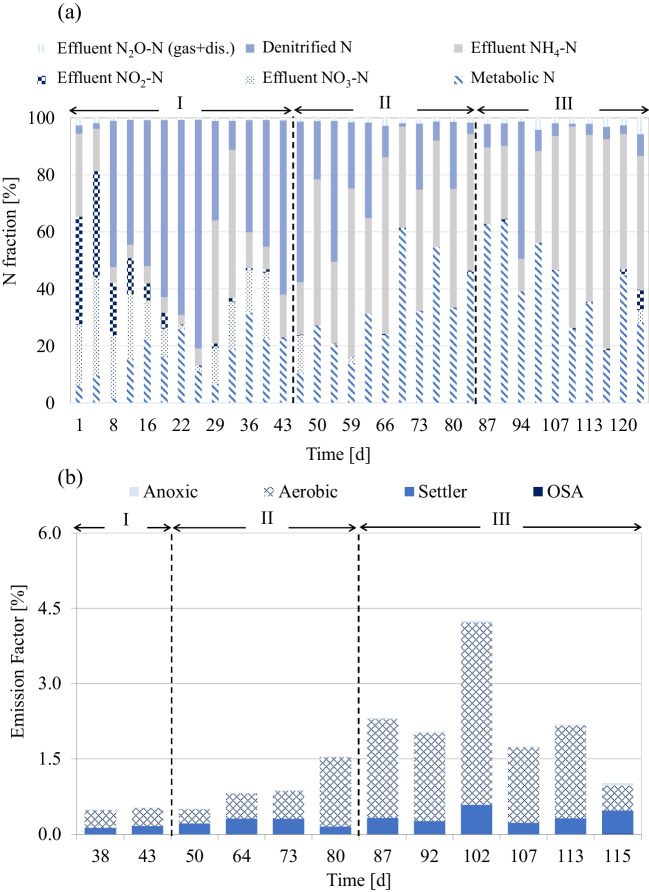
N fractions for each experimental period (**a**) and N_2_O emission factors (**b**)

Concerning TN removal, the highest removal efficiency (65% ± 19) was achieved in period I, compared to period II (58% ± 15) and period III (42% ± 14). Indeed, data reported in Fig. 2c shows that the HRT increase in the ASSR (from 4 to 6 h in period II and period III, respectively) led to a decrease in the average nitrogen removal. This result might be related to the significant worsening of nitrification occurring after the implementation of the OSA configuration. Indeed, while in period I, very high nitrification performances were obtained (79% ± 17 of influent NH_4_-N was nitrified); in periods II and III, the nitrification efficiency strongly decreased (respectively 48% and 27%, as average). Regarding the influence of OSA on nitrification performance, previous literature provides contrasting results. Wang et al. (2020) obtained an improvement of nitrification under OSA configuration due to the longer SRT favoring the growth of autotrophic bacteria. In contrast, other authors, such as Zhou et al. (2015), highlighted a reduction of nitrification efficiency related to the stress exerted to autotrophic biomass due to the exposure to anaerobic conditions. Moreover, since the sludge coming from the anaerobic reactor was recycled into the anoxic reactor, the decrease of nitrification efficiency could be likely related to the prolonged exposure to non-aerated conditions. This condition could have compromised the growth of autotrophic bacteria (the respirometric batch tests, described below, corroborated this result). The stress effect exerted by unaerated conditions was emphasized when the HRT in the anaerobic reactor was increased to 6 h, with nitrification efficiency that dramatically decreased to 27%. In Fig. 3, the nitrogen mass balance throughout experiments (a) and the N_2_O emission factors (b) are shown.

Figure 3a shows that during the experimental periods, nitrogen transformation was influenced by the operating conditions. Specifically, during period I, most of the influent nitrogen was denitrified (on average 43%). During periods II and III, a failure of nitrification occurred, as discussed below, due to stress conditions for nitrifying bacteria, which led to a significant increase of effluent ammonia concentration (on average from 5.5 mg L^−1^ for period I to 14.1 and 19.6 mg L^−1^ for period II and period III, respectively). During periods II and III, the metabolic N consumption increased (on average from 16% for period I to 38.2% and 54.2% for periods II and period III, respectively). The observed result could be related to the increase of the average influent COD concentration (and consequently of the influent BOD) observed in periods II and III. It is worth noting that the different operating conditions also influenced greenhouse gas emissions in terms of N_2_O-N. As shown in Fig. 3a, the N_2_O-N fraction (over the influent total nitrogen) was equal to 1%, 1.55%, and 3.54%, respectively for periods I, II, and III. This result may have relevant environmental implications since N_2_O-N has a global warming potential (GWP) significantly higher compared to CO_2_ (IPCC 2021). For completeness, in Fig. 3b, the pattern of N_2_O-N emission factor (EF_N2O_) in the three experimental periods is shown for each reactor. The aerobic reactor contributed with the highest EF_N2O_ throughout experiments and this result could be related to air supply. On average, the EF_N2O_ contribution of the aerobic reactor rose from 64% in period I to 76% in periods II and III, thus suggesting an increasing trend of N_2_O-N production during nitrification, especially under stress conditions. This result slightly contrasts with the current literature, which demonstrated, by using a life cycle approach (Liu et al. 2021), that the implementation of OSA configuration might reduce the amount of GHG emission by 23% (Liu et al. 2021). However, Liu et al. (2021), for their analysis, adopted a life cycle approach without considering measured data, thus underlying the importance of GHG monitoring during OSA operation for identifying the trade-off among effluent quality, reduction of sludge production (and consequently operational costs), and environmental impacts.

The OSA implementation promoted a significant increase of PO_4_-P removal, mainly noticed in periods III (50% ± 24) and II (48% ± 14), compared to period I (39% ± 24). Since authors did not observe the precipitation of any complex, this increase of PO_4_-P removal could be likely imputable to the presence of PAOs organisms that, under the alternate aerobic–anaerobic conditions promoted by OSA, can accumulate phosphorus (Mannina et al. 2017). Moreover, those results agree with Chudoba et al. (1992), who affirmed that in an OSA system, phosphate removal might be expected up to about 50%.

### Excess sludge production

**Fig. 4:**
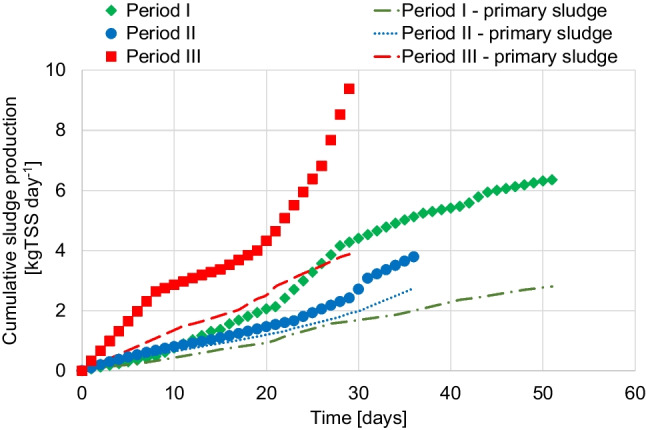
Cumulative sludge production and primary sludge results for each period

Figure 4 shows the trend profile of cumulative excess sludge production during experiments 4. It is worth noting that no settling of raw wastewater was carried out before biological treatments; therefore, the overall excess sludge production included the settleable solids contained in the raw wastewater (“primary” sludge). The biological and primary sludge reduction rates are reported in Table 2. From Table 2, the percentage of primary sludge under the OSA configuration was higher compared to the CAS configuration. Since the implementation of the anaerobic reactor only influences the biological sludge production, without affecting the primary sludge, the latter resulted predominantly in the excess sludge production under OSA configuration. As reported in Fig. 4, a noteworthy decrease in excess sludge (equal to 43.5%) was noticed in period II compared to period I. In contrast, mainly due to the higher average influent COD concentration, coupled with the increased temperatures, which led to increased sludge withdrawals and lower SRTs, the sludge reduction in period III was much lower (8.7%) compared to what was achieved in period II.

**Table 2:** Observed yield coefficient values under the real temperature conditions (Y_obs,*T*_) and corrected with respect to the standard temperature of 20 °C (*Y*_obs,20_); percentage of sludge reduction with respect to *Y*_obs,20_; percentage of primary and secondary sludge with respect to the total amount at the end of each period

	Period					
	I		II		III	
	Average	SD	Average	SD	Average	SD
*Y*_obs,*T*_ [gTSS/gCOD]	0.58	0.16	0.31	0.05	0.43	0.02
*Y*_obs,20_ [gTSS/gCOD]	0.46	0.18	0.26	0.23	0.42	0.21
*T* [°C]	11.6	1.8	13.8	2.3	18.6	1.8
Sludge reduction [%]	-	-	43.5	-	8.7	-
Primary sludge [%]	44	-	72	-	53	-
Biological sludge [%]	56	-	28	-	47	-

Moreover, as shown in Fig. 4, there was a lower difference between total and primary sludge in period II, meaning that the biological sludge was significantly reduced.

Regarding the observed yield coefficient (*Y*_obs_) values, summarized in Table 2, in period II, the *Y*_obs_ decreased from 0.58 to 0.31 gTSS g^−1^ COD, highlighting a reduction rate of 46.5% due to the OSA implementation, with an HRT of 4 h. However, in period III, this configuration demonstrated a lower decrease (0.58 to 0.43 gTSS g^−1^ COD) likely related to the higher average TSS concentration in the reactors coupled to the different operational features (Table 3). From the achieved results, the OSA configuration showed a lower production of excess sludge in both periods II and III, thus suggesting that sludge minimization can be successfully achieved. Nevertheless, the performance of OSA configuration can be significantly influenced by the plant operational conditions, which mainly depend on raw wastewater characteristics.

**Table 3:** Average values of total suspended solid concentrations [gTSS L^−1^] in the reactors throughout experiments

Section	Period					
	I		II		III	
	Average	SD	Average	SD	Average	SD
Anoxic	3.16	1.2	3.85	0.9	6.21	1.3
Aerobic	3.04	1.3	3.86	0.9	6.23	1.2
OSA	-	-	5.45	1.0	8.18	1.3

### Sludge settling properties and EPS content and composition

**Fig. 5:**
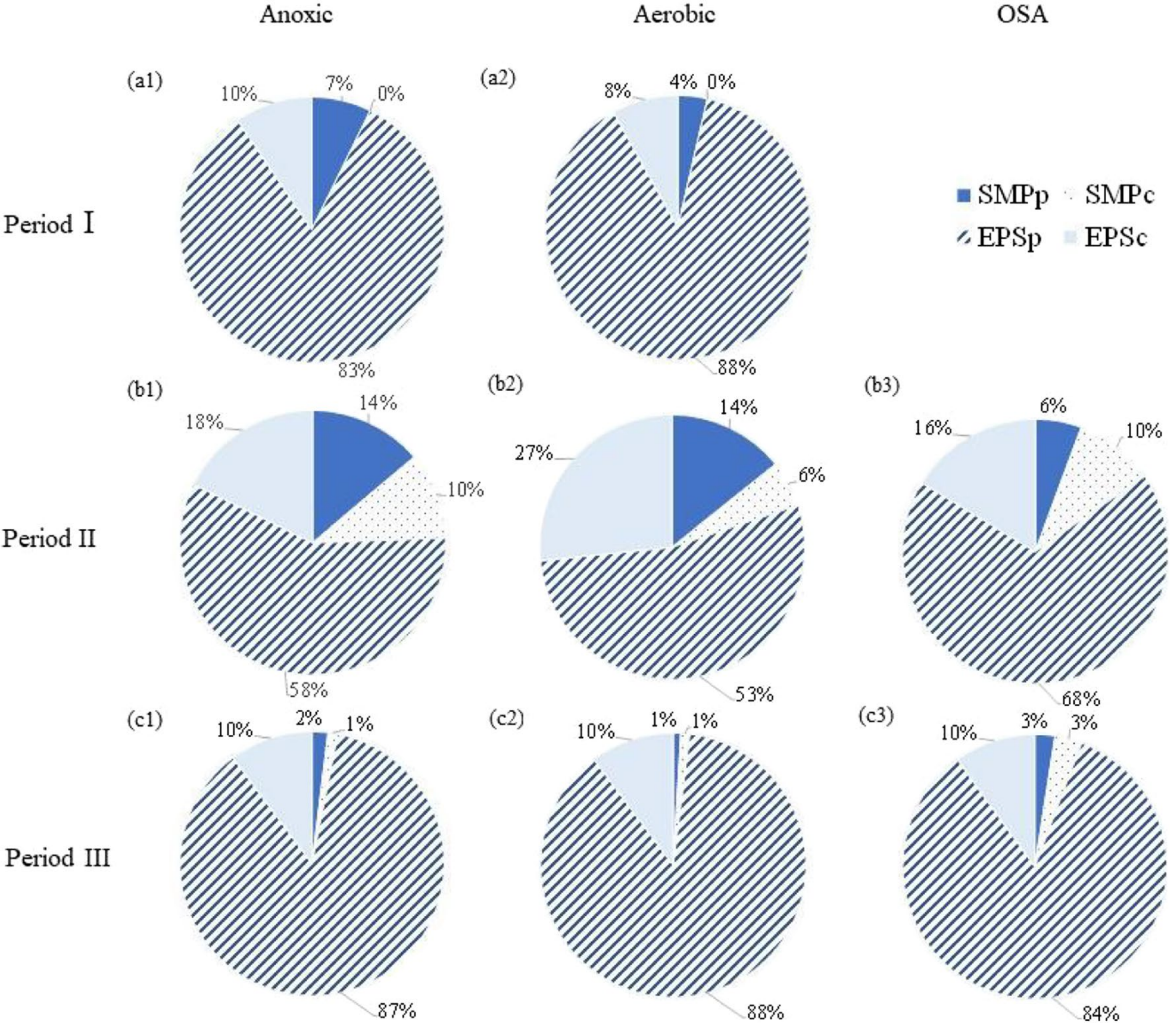
Specific EPS content and composition in period I (**a**), period II (**b**), and period III (**c**)

With the aim to highlight the effect of plant configuration on sludge physical features, the SVI and the EPS content were periodically measured. Figure 5 shows the SMP and EPS composition, expressed as proteins and carbohydrates percentage, in the anoxic (Fig. 5a), aerobic (Fig. 5b), and OSA (Fig. 5c) reactors. Table 4 summarizes the specific EPS and SMP concentrations (average values) in the different periods.

**Table 4:** Specific concentrations of SMP and EPS in each period for SMP and EPS for proteins and carbohydrates

	Period					
	I		II		III	
	EPS specific concentrations [mg g^−1^ TSS]					
	Average	SD	Average	SD	Average	SD
SMPp	8.8	4.1	6.3	2.0	1.0	0.5
SMPc	0.0	0.0	5.2	2.1	1.1	0.8
EPSp	138.5	7.0	36.6	13.5	49.9	2.5
EPSc	14.2	1.5	12.0	3.6	5.9	0.3
**Total**	**161.5**	**-**	**60.2**	**-**	**57.9**	**-**

As shown in Fig. 5 and Table 4, the specific EPS average value (as the sum of proteins and carbohydrates) in period I was 161.5 mg EPS g^−1^ TSS in both anoxic and aerobic reactors. In contrast, in period II, a reduction of 63% was observed (down to 60.2 mg g^−1^ TSS), while in period III, the average reduction was 64% (57.9 mg g^−1^ TSS) compared to period I. Moreover, the highest difference in the EPS composition in the different reactors was obtained in period II, while in period III, a slight reduction of SMPp (5 to 2%) amount and a slight increase of SMPc (0 to 2%) occurred (Fig. 5). As noticeable from Fig. 5b, during period II, it was noticed a SMP increase in the reactors, thereby suggesting the occurrence of cell lysis and EPS destructuration as sludge reduction mechanisms.

These results are in line with previous literature, which highlights that in OSA processes it can be observed an EPS destruction compared to CAS systems (Semblante et al. 2016a). Nevertheless, in the present study, no significant EPS reduction was observed from period II to period III (63% and 64%, respectively), likely related to the operational conditions.

Concerning sludge settling properties, the average SVI value in period I (117.5 ± 18.0 mL g^−1^ TSS) highlighted good sludge settleability. In contrast, after the implementation of the OSA configuration, a progressive worsening of sludge settling was observed, with increasing SVI values to 146.5 ± 30.7 mL g^−1^ TSS and 166.8 ± 22.2 mL g^−1^ TSS in period II and period III, respectively. This result is in line with previous literature that emphasized the deterioration of sludge settleability with the retention time increase in the anaerobic reactor (Sun et al. 2020). The worsening of sludge settling properties could be related to the EPS decrease observed during experiments; indeed, the EPS decrease, which is connected to bacterial substrate consumption under fasting conditions, likely promoted a destructuration of activated sludge floc structure, thus promoting a worsening of settling properties.

### Kinetic parameters assessed by respirometry

Table 5 summarizes the average values of heterotrophic and autotrophic parameters obtained in the different experimental periods; moreover, the level of significance (*p*-value) obtained by comparing the results achieved in CAS and OSA configurations between two consecutive periods are reported. According to the statistical analysis carried out, only with a *p*-value lower than 0.05, it was supposed that the parameter variation was affected by the OSA implementation in period II as well as the increase of HRT in the ASSR reactor in period III.

**Table 5:** Summary of the main heterotrophic kinetic and stoichiometric parameters (average values) and the results of significance level achieved with the statistical analysis (the standard deviation values in brackets)

Parameter	Symbol	Units	Heterotrophic				
			Period I	Period II		Period III	
			CAS	CAS-OSA	*p*-value	CAS-OSA	*p*-value
Max. growth yield	*Y*_*H*_	[gVSS g^−1^ COD]	0.457 ± 0.015	0.423 ± 0.029	0.025	0.415 ± 0.32	0.023
Decay rate	*b*_*H*_	[d^−1^]	0.59 ± 0.045	0.62 ± 0.002	0.039	0.82 ± 0.205	0.063
Max. growth rate	*μ*_*H*_	[d^−1^]	3.12 ± 0.61	2.67 ± 1.11	0.311	2.30 ± 0.88	0.725
Max. removal rate	*ν*_*H*_	[d^−1^]	7.09 ± 0.85	6.31 ± 2.64	0.021	6.00 ± 0.19	0.031
Net growth rate	*μ*_*H*_-*b*_*H*_	[d^−1^]	2.66 ± 0.522	2.06 ± 1.092	0.635	1.48 ± 0.560	0.710
Active fraction	*f*_*X*_	[%]	31.71 ± 0.21	43.44 ± 10.49	0.0004	33.20 ± 2.15	0.0004
Parameter	Symbol	Units	Autotrophic				
			Period I	Period II		Period III	
			CAS	CAS-OSA	*p*-value	CAS-OSA	*p*-value
Max. growth yield	*Y*_*A*_	[gVSS g^−1^ NH_4_-N]	0.22 ± 0.03	na	-	na	-
Decay rate	*b*_*A*_	[d^−1^]	0.12 ± 0.02	na	-	na	-
Max. growth rate	*μ*_*A*_	[d^−1^]	0.54 ± 0.17	na	-	na	-
Max. removal rate	*ν*_*A*_	[d^−1^]	3.28 ± 0.48	na	-	na	-
Nitrification rate	*N*_*R*_	[mgNH_4_ L^−1^ h^−1^]	5.55 ± 1.61	na	-	na	-

Concerning the maximum growth yield *Y*_*H*_, a general decrease was observed from period I through periods II and III, respectively; this variation was considered statistically significant (*p*-values equal to 0.025 and 0.023 comparing period I and period II, and period II and period III, respectively). These results demonstrated that, despite the significant fluctuations of the influent wastewater features, which could affect the operational parameters, the implementation of ASSR in the RAS line enabled to achieve a decrease in sludge production tendency. In period II, it was observed a moderate decrease in the maximum growth rate of heterotrophic bacteria *μ*_*H*_, while it was noticed an increase in the active fraction *f*_XH_ and an average increase of the endogenous decay rate *b*_*H*_. This result could indicate that in period II, the main mechanism for sludge reduction could be the uncoupled metabolism, rather than the heterotrophic endogenous decay (maintenance metabolism). The *b*_*H*_ and *f*_XH_ variation from period I to period II resulted statistically significant (*p*-value < 0.05). In period III, characterized by the HRT increase in the anaerobic reactor from 4 to 6 h, it was observed a significant increase in the endogenous decay rate (from 0.62 to 0.82 in periods II and III, respectively), coupled with a reduction of the active fraction (from 43 to 33% in periods II and III, respectively). These variations were statistically significant, with *p*-values < 0.05 and suggested that a main reduction mechanism could be due to the occurrence of endogenous metabolism, likely enhanced by the prolonged exposure to anaerobic conditions under substrate scarcity and the observed result is in line with previous findings (Chen et al. 2003).

**Fig. 6:**
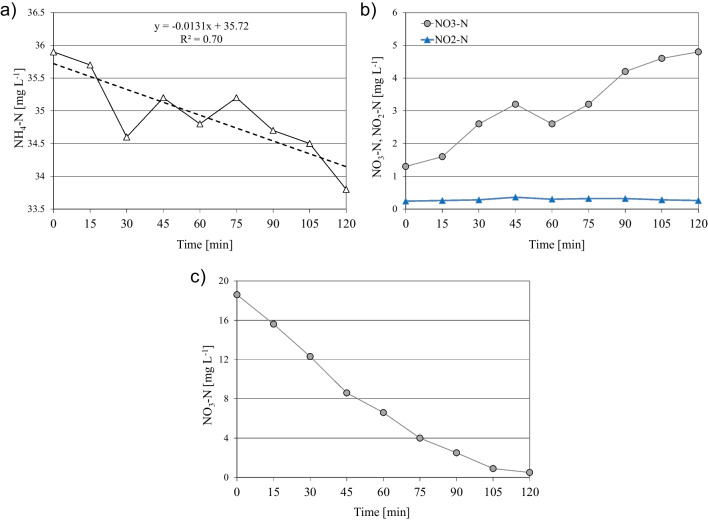
Examples of AUR tests during period I, with NH4-N (**a**) and NOX-N (**b**) trend profiles; NOX-N (**c**) trend profiles for the NUR test in period I

Regarding autotrophic species, respirometric batch tests in period I revealed an excellent activity of nitrifiers, with experimental values well in line with literature data (Capodici et al. 2016). In contrast, in periods II and III, a huge worsening of nitrifying bacteria activity was observed, likely related to the stress condition exerted by the prolonged exposure to anaerobic conditions, which made it impossible to achieve regular respirogram charts to be further processed to assess the kinetic and stoichiometric parameters. This result agreed with the huge worsening of nitrification observed in periods II and III, which was discussed above. The AUR test also confirmed these results. Indeed, as reported in Fig. 6, in period I, good nitrification development was observed, with no nitrite accumulation during the test (Fig. 6b). Concerning denitrification, the NUR tests showed good biomass behaviour in period I, with a nitrate uptake rate of 6.70 mgNO_3_-N g^−1^ VSS h^−1^, which suggested a good denitrifying ability in period I.

## Conclusions

The minimization of excess sludge was investigated by comparing a CAS system with a CAS-OSA configuration; the aim was to find a trade-off between sludge minimization, nitrogen removal, and resource recovery (PO_4_-P). The highest sludge minimization was achieved in period II, characterized by HRT in the ASSR of 4 h, with a reduction of 43.5% and without significantly compromising the effluent quality. On the other hand, the increased HRT in the anaerobic reactor to 6 h enabled high PO_4_-P removal but dramatically affected the system performance in terms of nitrification, sludge settling properties, and increased GHG emissions. Therefore, the findings of this manuscript help to identify a trade-off between sludge production, GHG emissions, and effluent quality when the OSA process is applied. Identifying suitable operating conditions is crucial to push towards emission reduction and plant carbon neutrality.

## Data Availability

All data and materials comply with field standards and are available to the authors.

## References

[CR1] APHA (2012) Standard methods for the examination of water and wastewater, 22nd edition edited by E. W. Rice, R. B. Baird, A. D. Eaton and L. S. Clesceri. American Public Health Association (APHA), American Water Works Association (AWWA) and Water Environment Federation (WEF), Washington, D.C.

[CR2] Cantekin C, Taybuga ES, Yagci N, Orhon D (2019) Potential for simultaneous nitrogen removal and sludge reduction of the oxic-settling-anaerobic process operated as a dual fed sequencing batch reactor. J Environ Manag 247:394–400. 10.1016/j.jenvman.2019.06.086 10.1016/j.jenvman.2019.06.086 31254755

[CR3] Capodici M, Fabio Corsino S, Di Pippo F, Di Trapani D, Torregrossa M (2016) An innovative respirometric method to assess the autotrophic active fraction: application to an alternate oxic-anoxic MBR pilot plant. Chem Eng J 300:367–375. 10.1016/j.cej.2016.04.134 10.1016/j.cej.2016.04.134

[CR4] Chen GH, An KJ, Saby S, Brois E, Djafer M (2003) Possible cause of excess sludge reduction in an oxic-settling-anaerobic activated sludge process (OSA process). Water Res 37(16):3855–3866. 10.1016/S0043-1354(03)00331-2 12909103 10.1016/S0043-1354(03)00331-2

[CR5] Chudoba P, Morel A, Capdeville B (1992) The case of both energetic uncoupling and metabolic selection of microorganisms in the OSA activated sludge system. Environ Technol 13(8):761–770. 10.1080/09593339209385207 10.1080/09593339209385207

[CR6] Collivignarelli MC, Canato M, Abba A, Miino MC (2019) Biosolids: what are the different types of reuse? J Clean Prod 238:117844 10.1016/j.jclepro.2019.117844

[CR7] Collivignarelli MC, Abbà A, Miino MC, Caccamo FM, Argiolas S, Bellazzi S, Bertanza G (2021a) Strong minimization of biological sludge production and enhancement of phosphorus bioavailability with a thermophilic biological fluidized bed reactor. Process Saf Environ Prot 155:262–276. 10.1016/j.psep.2021.09.026 10.1016/j.psep.2021.09.026

[CR8] Collivignarelli MC, Abbà A, Bertanza G, Baldi M, Setti M, Frattarola A, Carnevale Miino M (2021b) Treatment of high strength wastewater by thermophilic aerobic membrane reactor and possible valorisation of nutrients and organic carbon in its residues. J Clean Prod 280. 10.1016/j.jclepro.2020.124404

[CR9] Coma M, Rovira S, Canals J, Colprim J (2013) Minimization of sludge production by a side-stream reactor under anoxic conditions in a pilot plant. Biores Technol 129:229–235. 10.1016/j.biortech.2012.11.055 10.1016/j.biortech.2012.11.055 23247151

[CR10] DuBois M, Gilles KA, Hamilton JK, Rebers PA, Smith F (1956) Colorimetric method for determination of sugars and related substances. Anal Chem 28:350–356. 10.1021/ac60111a017 10.1021/ac60111a017

[CR11] Fazelipour M, Takdastan A, Borghei SM, Kiasat N, Glodniok M, Zawartka P (2021) Efficiency studies of modified IFAS-OSA system upgraded by an anoxic sludge holding tank. Sci Rep 11:1–14. 10.1038/s41598-021-03556-6 34921213 10.1038/s41598-021-03556-6 PMC8683438

[CR12] Ferrentino R, Langone M, Andreottola G (2021) Sludge reduction by an anaerobic side-stream reactor process: a full-scale application. Environ Chall 2:100016. 10.1016/j.envc.2020.100016 10.1016/j.envc.2020.100016

[CR13] Gardoni D, Ficara E, Fornarelli R, Parolini M, Canziani R (2011) Long term effects of the ozonation of the sludge recycling stream on excess sludge reduction and biomass activity at full-scale. Water Sci Technol 63(9):2032–2038 21902046 10.2166/wst.2011.456

[CR14] IPCC (2021) Sixth Assessment Report Working Group 1: The physical science basis. Technical summary. https://www.ipcc.ch/report/ar6/wg1/. Accessed 15 July 2024

[CR15] Jiang LM, Zhou Z, Niu T, Jiang L, Chen G, Pang H, Qiu Z (2018) Effects of hydraulic retention time on process performance of anaerobic side-stream reactor coupled membrane bioreactors: kinetic model, sludge reduction mechanism and microbial community structures. Bioresour Technol 267:218–226 30025317 10.1016/j.biortech.2018.07.047

[CR16] Jupp AR, Beijer S, Narain GC, Schipperc W, Slootweg JC (2021) Phosphorus recovery and recycling – closing the loop. Chem Soc Rev 50:87–101 33210686 10.1039/D0CS01150A

[CR17] Karlikanovaite-Balikci A, Yagci N (2019) Evaluation of sludge reduction in an oxic-settling-anoxic system operated with step feeding regime for nutrient removal and fed with real domestic wastewater. J Environ Manag 243:385–392. 10.1016/j.jenvman.2019.05.042 10.1016/j.jenvman.2019.05.042 31103684

[CR18] Le-Clech P, Chen V, Fane TAG (2006) Fouling in membrane bioreactors used in wastewater treatment. J Memb Sci 284:17–53. 10.1016/j.memsci.2006.08.019 10.1016/j.memsci.2006.08.019

[CR19] Liu X, Iqbal A, Huang H, Zan F, Chen G, Wu D (2021) Life cycle assessment of deploying sludge minimization with (sulfidogenic-) oxic-settling-anaerobic configurations in sewage-sludge management systems. Biores Technol 335:125266 10.1016/j.biortech.2021.125266 34020155

[CR20] Lowry OH, Rosebrough NJ, Farr L, Randall R (1951) Protein measurement with the Folin phenol reagent. J Biol Chem 193:265–275. 10.1016/0304-3894(92)87011-4 14907713 10.1016/0304-3894(92)87011-4

[CR21] Mannina G, Mineo A (2023) Polyhydroxyalkanoate production from fermentation of domestic sewage sludge monitoring greenhouse gas emissions: aa pilot plant case study at the WRRF of Palermo University (Italy). J Environ Manag 348:119423 10.1016/j.jenvman.2023.119423 37871545

[CR22] Mannina G, Morici C, Cosenza A, Di Trapani D, Ødegaard H (2016) Greenhouse gases from sequential batch membrane bioreactors: a pilot plant case study. Biochem Eng J 112:114e122. 10.1016/j.bej.2016.04.010 10.1016/j.bej.2016.04.010

[CR23] Mannina G, Ekama GA, Capodici M, Cosenza A, Di Trapani D, Ødegaard H (2017) Moving bed membrane bioreactors for carbon and nutrient removal: the effect of C/N variation. Biochem Eng J 125:31–40. 10.1016/j.bej.2017.05.005 10.1016/j.bej.2017.05.005

[CR24] Mannina G, Ekama GA, Capodici M, Cosenza A, Di Trapani D, Ødegaard H, van Loosdrecht MCM (2018) Influence of carbon to nitrogen ratio on nitrous oxide emission in an Integrated Fixed Film Activated Sludge Membrane BioReactor plant. J Clean Prod 176:1078–1090. 10.1016/j.jclepro.2017.11.222 10.1016/j.jclepro.2017.11.222

[CR25] Mannina G, Badalucco L, Barbara L, Cosenza A, Di Trapani D, Gallo G, Laudicina VA, Marino G, Muscarella SM, Presti D (2021a) Enhancing a transition to a circular economy in the water sector: the EU project wider uptake. Water 13:946. 10.3390/w13070946 10.3390/w13070946

[CR26] Mannina G, Alduina R, Badalucco L, Barbara L, Capri FC, Cosenza A, Di Trapani D, Gallo G, Laudicina VA, Muscarella SM, Presti D (2021b) Water resource recovery facilities (WRRFS): the case study of Palermo university (Italy). Water 13:3413. 10.3390/w13233413 10.3390/w13233413

[CR27] Mannina G, Pandey A, Sirohi R (2022) Smart solutions for wastewater: road-mapping the transition to circular economy. In: Current developments in biotechnology and bioengineering. Elsevier. 10.1016/C2021-0-00564-2

[CR28] Mannina G, Barbara L, Cosenza A, Wang Z (2023) Treatment and disposal of sewage sludge from wastewater in a circular economy perspective. Curr Dev Biotechnol Bioeng 11–30. 10.1016/B978-0-323-99920-5.00011-1

[CR29] Martins CL, Velho VF, Magnus BS, Xavier JA, Guimarães LB, Leite WR, Costa RHR (2020) Assessment of sludge reduction and microbial dynamics in an OSA process with short anaerobic retention time. Environ Technol Innov 19:101025. 10.1016/j.eti.2020.101025 10.1016/j.eti.2020.101025

[CR30] Morello R, Di Capua F, Esposito G, Pirozzi F, Fratino U, Spasiano D (2022) Sludge minimization in mainstream wastewater treatment: mechanisms, strategies, technologies, and current development. J Environ Manag 319:115756. 10.1016/j.jenvman.2022.115756 10.1016/j.jenvman.2022.115756 35982561

[CR31] Saby S, Djafer M, Chen GH (2003) Effect of low ORP in anoxic sludge zone on excess sludge production in oxic-settling-anoxic activated sludge process. Water Res 37(1):11–20. 10.1016/S0043-1354(02)00253-1 12465783 10.1016/S0043-1354(02)00253-1

[CR32] Semblante GU, Hai FI, Bustamante H, Guevara N, Price WE, Nghiem LD (2016a) Biosolids reduction by the oxic-settling-anoxic process: Impact of sludge interchange rate. Biores Technol 210:167–173. 10.1016/j.biortech.2016.01.010 10.1016/j.biortech.2016.01.010 26810193

[CR33] Semblante GU, Hai FI, Bustamante H, Price WE, Nghiem LD (2016b) Effects of sludge retention time on oxic-settling-anoxic process performance: biosolids reduction and dewatering properties. Biores Technol 218:1187–1194. 10.1016/j.biortech.2016.07.061 10.1016/j.biortech.2016.07.061 27474952

[CR34] Sun Z, Li M, Wang G, Yan X, Li Y, Lan M, Liu R, Li B (2020) Enhanced carbon and nitrogen removal in an integrated anaerobic/anoxic/aerobic-membrane aerated biofilm reactor system. RSC Adv 10:28838–28847. 10.1039/d0ra04120c 35520069 10.1039/d0ra04120c PMC9055795

[CR35] Tchobanoglous G, Burton FL, Stensel HD (2003) Wastewater engineering: treatment and reuse, 4th edn. Metcalf and Eddy Inc. McGraw-Hill Higher Education, New York

[CR36] Tsuneda S, Mikami M, Kimochi Y, Hirata Y (2005) Effect of salinity on nitrous oxide emission in the biological nitrogen removal process for industrial wastewater. J Hazard Mater B119:93–98 10.1016/j.jhazmat.2004.10.025 15752853

[CR37] Vitanza R, Cortesi A, De Arana-Sarabia ME, Gallo V, Vasiliadou IA (2019) Oxic settling anaerobic (OSA) process for excess sludge reduction: 16 months of management of a pilot plant fed with real wastewater. J Water Process Eng 32:100902. 10.1016/j.jwpe.2019.100902 10.1016/j.jwpe.2019.100902

[CR38] Wang K, Zhou Z, Zheng Y, Jiang J, Huang J, Qiang J, An Y, Jiang L, Jiang LM, Wang Z (2020) Understanding mechanisms of sludge in situ reduction in anaerobic side-stream reactor coupled membrane bioreactors packed with carriers at different filling fractions. Bioresour Technol 316:123925. 10.1016/j.biortech.2020.123925 32758921 10.1016/j.biortech.2020.123925

[CR39] Ye FX, Zhu RF, Li Y (2008) Effect of sludge retention time in sludge holding tank on excess sludge production in the oxic-settling-anoxic (OSA) activated sludge process. J Chem Technol Biotechnol 83:109–114. 10.1002/jctb.1781 10.1002/jctb.1781

[CR40] Zhang R, Mao Y, Meng L (2021) Excess sludge cell lysis by ultrasound combined with ozone. Sep Purif Technol 276:119359. 10.1016/j.seppur.2021.119359 10.1016/j.seppur.2021.119359

[CR41] Zhang C, Guisasola A, Baeza JA (2022) A review on the integration of mainstream P-recovery strategies with enhanced biological phosphorus removal. Water Res 212:118102. 10.1016/j.watres.2022.118102 35091221 10.1016/j.watres.2022.118102

[CR42] Zhou Z, Qiao W, Xing C, An Y, Shen X, Ren W, Jiang L, Wang L (2015) Microbial community structure of anoxic-oxic-settling-anaerobic sludge reduction process revealed by 454-pyrosequencing. Chem Eng J 266:249–257

